# Unequal ageing: the quality of life of senior citizens in the EU before and after COVID-19. A multidimensional approach

**DOI:** 10.3389/fpubh.2025.1506006

**Published:** 2025-01-29

**Authors:** Elżbieta Roszko-Wójtowicz, Klaudia Przybysz, Agnieszka Stanimir

**Affiliations:** ^1^Department of Economic and Social Statistics, Faculty of Economics and Sociology, University of Łódź, Łódź, Poland; ^2^Department of Econometrics and Operational Research, Faculty of Economics and Finance, Wrocław University of Economics and Business, Wrocław, Poland

**Keywords:** quality of life of seniors, economic inequality, health disparities, synthetic measure of senior quality of life (SMSQoL), Hellwig’s method, COVID-19 pandemic, European Union

## Abstract

**Introduction:**

The ageing population presents a significant demographic and socio-economic challenge for the European Union (EU). Declining fertility rates, coupled with increasing life expectancy, have led to a growing proportion of older individuals within the population, raising concerns about their quality of life. This study aims to assess the quality of life for seniors across EU countries in the years 2015, 2019, and 2022, with a particular focus on the impact of the COVID-19 pandemic. The research seeks to answer the following question: How has the quality of life among seniors in the EU evolved over time, and how has the COVID-19 pandemic affected this trajectory? We hypothesize that the pandemic has exacerbated existing socio-economic inequalities, particularly affecting the most vulnerable older populations.

**Methods:**

This study utilises the Synthetic Measure of Senior Quality of Life (SMSQoL) to evaluate the living conditions of seniors across four critical domains: health, finances, social relations, and environment. Data for the analysis were drawn from Eurostat and national statistical reports, complemented by pilot studies conducted in selected EU countries. The pilot studies focused on gathering qualitative data to supplement the quantitative measures, especially in areas where standardised data were incomplete or unavailable. The assessment spans three years: 2015 (pre-pandemic baseline), 2019 (immediate pre-pandemic), and 2022 (post-pandemic). The analysis includes 27 EU member states and uses both descriptive and inferential statistical methods to evaluate trends and disparities. Cross-sectional analysis was applied to examine the impact of differing social policies, levels of social security, access to healthcare, and economic strength across these countries.

**Results:**

The analysis reveals significant disparities in the quality of life among seniors across EU countries, with pronounced differences between regions. In particular:

- Northern and Western Europe: seniors in these regions generally experienced improved overall life quality over the observed period. Countries such as Sweden and Germany reported gains in health, financial stability, and social relations, largely due to strong social policies and robust healthcare systems.
- Eastern Europe: Seniors in Eastern European countries, including Romania and Bulgaria, continued to face substantial challenges, with minimal improvements in quality of life. Economic instability, limited access to healthcare, and weaker social security systems were identified as key contributors to this stagnation.
- Impact of COVID-19: the pandemic exacerbated existing inequalities, particularly in the domains of social relations and finances. Seniors in economically vulnerable regions were disproportionately affected by social isolation and reduced income, intensifying the pre-existing challenges in their living conditions.

Quantitative analysis confirmed that while some regions showed resilience, the most vulnerable populations experienced a sharp decline in their overall quality of life, particularly between 2019 and 2022.

**Discussion:**

The findings from this study highlight the persistence of economic and social inequalities in the life conditions among seniors across the EU. While countries in Northern and Western Europe have made strides in improving senior living conditions, Eastern Europe continues to face significant challenges. The COVID-19 pandemic acted as a catalyst, exacerbating these inequalities, particularly in terms of social isolation and financial insecurity. These results align with previous studies that have highlighted the uneven impact of social policies and economic strength on senior well-being across Europe. The disparities underscore the need for more balanced and equitable policy interventions that can address the vulnerabilities of older populations, particularly in regions struggling with economic instability. Future research should focus on longitudinal studies that track the recovery trajectories of seniors post-pandemic and assess the effectiveness of policy measures aimed at mitigating these disparities.

## 1 Introduction

The concept of unequal ageing has become increasingly relevant in the context of the European Union (EU), where economic inequality plays a significant role in shaping the experiences of older populations across different member states. Ageing within the EU is not a uniform process; instead, it is deeply influenced by the socio-economic conditions prevailing in each country. This divergence, commonly referred to as economic disparity, manifests itself not only in differences in pension and retirement benefits but also in the varying levels of access to healthcare, the quality of medical services, and the availability of activities and social programmes tailored for seniors. The issue of quality of life for older populations is therefore critical in the context of preventing social inequalities within the EU. As a key area for addressing these inequalities, seniors’ standard of living is often used to measure the effectiveness of actions taken in social policy, healthcare, and social integration (1). With the proportion of senior citizens rising due to longer life expectancy and declining fertility rates, their impact on various facets of social and economic life intensifies (2, 3). Understanding and addressing the disparities in the living conditions of this growing population is not only a matter of social justice but also essential for ensuring the sustainability of the EU’s social and economic systems.

This study aims to conduct a multidimensional comparison of the living situations of seniors across EU countries in the years 2015, 2019, and 2022. The research is guided by three specific objectives:

1. To identify the determinants of the quality of life for seniors in the EU.
2. To construct a Synthetic Measure of Senior Quality of Life (SMSQoL) and sub-measures across four domains: health, finances, social relations, and environment for identifying areas of inequality.
3. To assess the impact of the COVID-19 pandemic on the living situation of seniors in the EU.

The analysis is grounded in the hypothesis that the quality of life in the case of seniors is not uniformly distributed across the EU, with substantial economic stratifications persisting over time. The significance of the financial dimension in shaping the overall standard of living for seniors has been demonstrated by several authors. For example, Kim and Um (4) highlights that the stress associated with financial difficulties adversely affects seniors’ quality of life. Similarly, Kwon et al. (5) indicate that factors influencing the quality of life among older women in Korea vary depending on their economic status. Huang et al. (6) have also explored the impact of financial conditions on seniors’ perceptions of their standard of living. In this paper, we adopt a different perspective. Recognising the central role of financial factors in studies on seniors’ quality of life, we investigate whether the financial situation of seniors within the European Union is improving. In light of EU policies aimed at reducing disparities, our research hypotheses 1 and 2 address a particularly critical issue.

Specifically, we hypothesise:

H1: The living situation of seniors in the EU improved most notably in the financial domain during the years studied.

H2: The living conditions of seniors are heterogeneous across the EU, with persistent inequalities that have not diminished over the years.

H3: The COVID-19 pandemic has led to a deterioration in senior living conditions, particularly within specific domains.

From this point of view, the study of the living situation of seniors should not only be a one-off analysis at a specific point in time, but it ought to be repeated, comparisons should be made and the directions in which possible changes are taking place should be monitored (7). Such an approach allows for active and objective observation of the situation of seniors, emerging inequalities, and makes it possible to react quickly by selecting appropriate social policy tools or introducing changes in the senior programmes offered.

The study uses data from Eurostat and national reports, supplemented by two pilot studies conducted in 2020 and 2024, to explore the subjective and objective determinants of senior quality of life. These determinants are categorised into four domains: health, finances, social relations, and environment, which were identified by seniors themselves as the most important areas affecting their well-being.

The methodology employed involves the application of Z. Hellwig’s development pattern method to construct the synthetic measure and rank countries based on the quality of life of their senior populations. By comparing the SMSQoL across the selected years, the study provides insights into the changes and trends in senior living conditions within the EU. The analysis further categorises countries into four groups based on their performance in the synthetic measure, illustrating the significant disparities between them.

The findings reveal that while some EU countries, particularly in Northern and Western Europe, have consistently provided high living standards for seniors, others, especially in Eastern Europe, have lagged, resulting in pronounced economic disparities. These disparities have been exacerbated by the COVID-19 pandemic, particularly in the domains of social relations and finances, highlighting the need for targeted policy interventions to address the unique challenges faced by seniors in different regions.

This study contributes to the ongoing discussion on economic inequality within the EU, particularly in the context of ageing populations. By providing a comprehensive analysis of the factors that contribute to the quality of life for seniors, this research offers valuable insights for policymakers seeking to reduce inequalities and improve the overall well-being of older citizens across the EU.

The significance of our study within the current body of knowledge lies in its comprehensive approach to measuring quality of life. Firstly, we incorporate domains identified by seniors themselves, through pilot studies, as crucial to their life circumstances. Subsequently, we align these domains with relevant indicators derived from data published by Eurostat. The classification of EU countries based on the values of the synthetic measure we constructed enabled us to identify disparities not only in overall living standards but also at the domain level. This is particularly relevant to the European Union’s ongoing efforts to reduce disparities among the Member States.

This article is divided into five sections: introduction, literature review, materials and methods, results, conclusions and discussion. The introduction sets the research aims and questions. The literature review provides background on the quality of seniors’ life and discussion on definitions of this issue. The methodology section details our research scope and methods. The results section describes our findings.

## 2 Literature review

### 2.1 Quality of life

In approaching the discussion on the assessment of quality of life, it is important to emphasise the lack of universality concerning this issue due to different approaches existing in the literature. The World Health Organisation Quality of Life Assessment Group (WHOQOL) (100, p. 1405) defined QoL as an individual’s perception of his or her position in life in the context of the culture and value systems in which he or she lives, and in relation to his or her goals, expectations, standards and concerns. This concept, in a complex way, is influenced by physical health, mental state, level of independence, social relationships, personal beliefs and the individual’s relationship to the salient features of his or her environment. It is a multidimensional definition that includes subjectively perceived physical, psychological and social dimensions, as well as general age-and culture-specific perceptions of quality of life and health (8, 9). The concept of quality of life is widely used in research (10, 11). An important aspect seems to be, however, the discussion of which dimensions of QoL gain or lose importance in later life (12, 13). Because of the changes of different nature (biological, psychological and social) that occur in life, ageing needs to be understood in a multidimensional way (14–16). Zhang et al. (17) point to the existence of a link between inequalities and health, which results from the accumulation of adverse social circumstances throughout life, leading to negative health outcomes for older adults. Similarly, Niedzwiedz et al. (101, p. 368) argue that socio-economic inequalities affecting quality of life stem from disparities that “get under the skin” and become more pronounced in old age. However, Zhang et al. (17) also highlight the “age-as-leveler” phenomenon, where, with advancing age – particularly among seniors – socioeconomic differences in health tend to diminish due to the levelling of disparities in pensions and healthcare. Therefore, it is crucial to assess this issue through the lens of health, taking into account the socio-economic resources and inequalities among seniors. Zhang et al. (17) advocate for research on inequalities affecting older adults to consider not only health but also socio-economic status, which both determines individual inequalities and is shaped by the environment in which individuals live. Positive changes are associated, for example, with gaining more life experiences or increased time spent with grandchildren and family. Older people also have more time to actively participate in leisure activities. Negative changes, on the other hand, mainly relate to the loss of a partner, the onset of illness and related dysfunctions or the need to care for an ill or disabled partner. The changes described have an indisputable impact on seniors’ quality of life. However, the strength of this impact will depend on a number of factors such as personality, resilience, having social support, physical condition, satisfaction with one’s health or acquired coping skills in life (18–20).

Authors of many publications define quality of life quite often from the point of view of successful ageing (21). In accordance with this view, the most important factors will be primarily physical well-being understood as the absence of physical impairment (22, 23) and mental well-being that ensures the continuity of cognitive functions (24, 25). These enable seniors to continue to participate actively in daily life (22, 23, 26). A high correlation between the presence of different types of medical conditions and seniors’ level of quality of life assessment is often indicated (27, 28). Some studies point to the key role of the subjective assessment of one’s life on the overall quality of life score, and its superiority over objective socio-economic indicators (14, 29). In turn, this subjective assessment is influenced not only by the factors mentioned above, but also by social or daily activities through which the senior can sustain a sense of agency (30). Free time is also an important factor in the discussion on seniors’ standard of living. Leisure time is an attribute associated with the later period in life. In many studies, seniors emphasise that the freedom to decide how to spend their free time is very important for them (31–33).

The issue of quality of life in relation to older populations is not only important from a subjective point of view. It is often a yardstick for measuring the effectiveness of social policy in the area of preventing inequalities, healthcare or social inclusion activities. Hence, there are numerous tools to measure QoL, such as the World Health Organisation Quality of Life Instrument (WHOQOL), the 5-dimensional EuroQOL (EQ-5D), the Quality of Life, Enjoyment and Satisfaction Questionnaire (Q-LES), the Long-Term Care Quality of Life Assessment Scale (LTC-QOL), and the Satisfaction with Life Scale (SWLS) (1).

**Figure 1:**
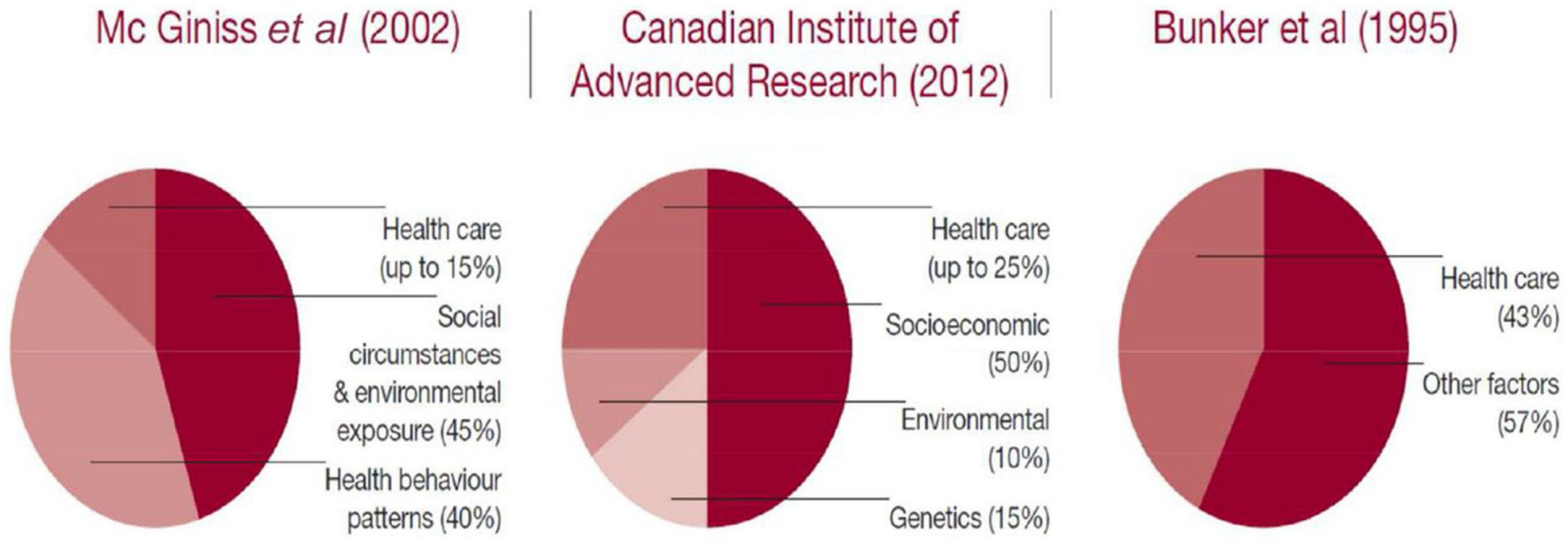
Estimates of the contribution of the main drivers of health status. Reprinted with permission from Donkin et al. (35), licensed under CC BY-NC 3.0 IGO.

The Social Determinants of Health framework (34) highlights the influence of structural factors—such as income, employment, education, and social capital—on health and well-being. For instance, individuals with higher socio-economic status typically enjoy better access to healthcare, nutritional resources, and social support networks, all of which contribute to healthier ageing. Conversely, systemic barriers experienced by lower-income populations exacerbate chronic health conditions, reduce life expectancy, and diminish quality of life in old age. Although there are differing opinions on the extent of the impact of social determinants on health (SDH), the report compiled by the Kings Fund (Figure 1) highlights that social and environmental factors play a crucial role, accounting for between 45 and 60% of the variation in health outcomes (35).

This study builds upon the quality of life framework, integrating insights from the Cumulative Disadvantage Theory (36), which underscores how inequalities accumulate over time, resulting in diverging health and socio-economic outcomes later in life. The findings presented here make a significant contribution by elucidating the factors that mediate the effects of social stratification and health inequalities. These insights are particularly critical for understanding and addressing the challenges faced by older adults. By identifying the structural causes of unequal ageing, this research provides a foundation for developing policies aimed at reducing widening inequalities and improving health outcomes across the socio-economic spectrum.

### 2.2 Domains

Previous research on seniors’ quality of life and its determining factors has concentrated on various areas often referred to as domains. The concept of domains was used by Brown et al. (37) in the context of areas that are important to seniors as opposed to people from younger cohorts. Those were [(37), p. 240]: family, social and leisure activities, health, living conditions, and religion. In contrast, Netuveli and Blane (38) based on a review of the literature, detailed three main domains:

- Physical health, general (i.e., self-rated health) or disease-specific (i.e., Asthma).
- Psychological (i.e., subjective well-being, happiness and life satisfaction).
- Social (i.e., social relationships and networks).

In the very common study of quality of life using the WHOQOL-BREF questionnaire, the concept of four basic domains for assessing quality of life is operationalised, including physical, mental and social relations along with the environment (39, 40). Also the bottom-up spillover theory, often used to measure the overall quality of life (41), claims that the overall quality of life is affected by a variety of life domains, such as health, social life, leisure life and emotional well-being (42, 43).

Numerous authors emphasise the health domain, encompassing both physical and mental aspects, as the most important factor in evaluating seniors’ well-being (44–46). In contrast, the relationship between physical activity and health, and its subsequent positive impact on overall life satisfaction, is demonstrated by Cabak et al. (47), as well as Szychowska and Drygas (48). A different approach to the health-related domain is presented by authors investigating the impact of active ageing on the health of seniors and, by extension, on improving their quality of life (49–53).

The European Union’s calls for the inclusion of all social groups, especially seniors, give the social relations domain a great deal of importance. Its impact on the assessment of quality of life has been studied by many authors (54–56). The issue of the impact of social networks on the well-being and health of older people is presented in Guadalupe and Vicente (57). An important aspect of well-being, particularly for seniors, is related to financial issues. Several studies have highlighted the influence of the financial domain on seniors’ life satisfaction (58–60). The economic situation also affected seniors’ well-being during the COVID-19 pandemic restrictions (61). It has been noted that seniors in financially precarious conditions faced a so-called double lockdown (62).

### 2.3 COVID-19

The impact of the COVID-19 pandemic on the quality of life of individuals, including the older adults, is indisputable. However, it is known that it can affect different areas of life. Numerous researchers have devoted attention to examining the impact of pandemic COVID-19 on the quality of life of seniors. Some have focused on the psychological and social well-being of seniors in light of the strictures associated with the pandemic (63–65), while others have investigated its impact on seniors’ decreased mobility associated with physical inactivity and sedentary lifestyles (66). An important aspect raised by Buffel et al. (62) is the widening of social inequalities, the marginalisation of older people, especially those who are economically disadvantaged. The authors point out that economically disadvantaged seniors experienced a so-called double lockdown. This refers to social distance combined with an increase in social and spatial inequalities. In contrast, the impact of social isolation and feelings of loneliness on seniors’ quality of life was studied by Briere et al. (67).

Seniors who recovered from COVID-19 also faced a reduction in health-related quality of life through the negative impact of fatigue, pain, low physical activity and cognitive-communication problems caused by the disease (68). Social contacts are one of the pillars in the study of seniors’ quality of life (69). The effect of online courses on reducing loneliness in older people during the pandemic period was shown by Yang et al. (70).

Few studies have examined quality of life from the perspective of seniors (14, 71). Filling this research gap, we have focused our analysis on seniors and those factors that they themselves define as the most important to their well-being. An approach in which, through a pilot study, we obtain the responses of the seniors themselves concerning the important areas that influence their perception of their life situation, seems therefore expedient. We call these areas domains.

## 3 Data and methods

### 3.1 Pilot studies

Two pilot studies were conducted among seniors in southern Poland in January–March 2020 and the corresponding period in 2024. The first took place in early 2020, i.e., during the initial phase of the COVID-19 pandemic, which was also the reason for not continuing the study and stopping it in the pilot phase. It covered large, medium and small towns in South-West Poland and 57 respondents aged 60+. Respondents were selected using the snowball method, due to the need to control costs and the frequent reluctance of older people to complete the questionnaire. The method chosen allowed us to show the next survey participant that their predecessor had managed to complete the questionnaire. Respondents answered the questionnaire on various aspects of their lives. Some of the interviews conducted were in-depth interviews, so that it was possible to clarify doubts or respond to a respondent’s uncertainty in an area on an ongoing basis, as well as to observe the respondent’s emotions about areas of life that were important to them.

In 2024, the pilot study was repeated using the same questionnaire. Its main aim was to find out whether any differences were observed in the living situation of seniors after the COVID-19 pandemic and whether they had changed their attitudes regarding the importance of particular domains in their lives. The study this time, by design, was purely pilot and included 55 people. See Table 1 for a description of the profiles of respondents participating in both surveys.

**Table 1:** Respondents’ profiles.

	Demographic characteristics	2020		2024	
		Frequency	% of total	Frequency	% of total
Gender	Female	43	75	45	82
	Male	14	25	10	18
Age (years)	65–69	25	44	28	51
	70–74	15	26	15	27
	75 and above	17	30	12	22
Marital status	Single	28	51	32	58
	Not single	29	49	23	42
Level of education	Higher education	24	42	30	54
	Other	33	58	25	46
Place of residence	Small town	26	46	20	36
	Medium town	20	35	25	46
	Large city	11	19	10	18

Source: own elaboration, total number of respondents N_2020_–57; N_2024_–55.

### 3.2 Description of the domains and variables selected

The domains, detailed in the pilot study conducted, were not identified from a single question asked to respondents. When responding to various questions (asked most often on the Lickert scale) about the domains that were important to them, seniors most often indicated the ones used for this analysis. Four important areas were thus identified as the domains most important in the lives of seniors:

1. Health
2. Finance
3. Relationships
4. Environment

Some consistency can be seen here in terms of the areas assessed by the researchers as influencing seniors’ assessment of their quality of life. These are often described as health, material, social and emotional issues [see e.g., (9, 27)]. An advantage of our analysis is that the listed order of domains is determined by the respondents themselves in order from the most important to the least important. The appearance of the domain Environment came as somewhat of a surprise. Some studies analyse the impact of a country’s socio-economic situation on the functioning of seniors (72), but the discovery that seniors themselves perceive this as a key element in assessing their quality of life is an important finding of our study.

The next step of the study was to establish indicators representing each domain in every European Union country.

The Health domain is indicated by numerous authors (44, 46) as the most important in studies on seniors’ quality of life. As can be seen, they also identified this domain as the most important for their feelings of well-being. Both subjective and objective variables were used to represent the Health domain.

The impact of financial situation on living standards, not only for seniors, has been shown in many publications (59). Also in our pilot study, seniors indicated this domain as the second most important domain in terms of assessing their living situation. In this study, the Finances domain is represented by four variables.

The area of social activity for seniors is indicated as important for successful and healthy ageing (73). All programmes aimed at the social inclusion of seniors are based on their activation in this area. Our pilot study confirms the importance of this domain in the subjective assessment of seniors. In the Relations domain, five variables were used.

The Environment domain is designed to reflect the socio-economic and environmental conditions in which seniors live. For instance, it is posited that a high level of democracy correlates with increased social engagement among residents (74). Similarly, the influence of sustainable development—represented in this study by the consumer footprint variable—has been discussed by Clement et al. (75) and Leverenz-Soetaert (76). Furthermore, the relationship between socio-economic conditions and the standard of living for seniors has been widely examined in previous studies [e.g., (77, 78)]. However, the fact that seniors notice its importance in their own lives and are able to perceive its impact on their personal living situation is evidence of the increasing awareness of this social group. The following variables were used to describe the domain named Environment (see Table 2).

**Table 2:** Domain and variables.

Health	Variables	Finances	Variables
1. Healthy years of life at age 65 female	X_1_	1. Excessive burden of housing costs	X_7_
2. Healthy years of life at age 65 male	X_2_	2. Inability to make ends meet	X_8_
		3. Median net income among 65+	X_9_
3. Health rated as good or very good	X_2_		
		4. Inability to face unexpected financial expenses	X_9_
4. Self-reported unmet needs for medical examination	X_4_		
5. Projected life expectancy for 2030 female	X_5_		
6. Projected life expectancy for 2030 male	X_6_		

Relations	Variables	Environment	Variables
1. Overall satisfaction of relationships 65+	X_11_	1. Expenditure on pensions	X_16_
		2. Employment rate of older workers	X_17_
2. Internet use: never	X_12_		
		3. Consumer footprint	X_18_
		4. Poverty rate among 65+	X_19_
		5. Projected old-dependency ratio	X_20_
3. Internet use (65–74)	X_13_		
		6. Democracy index	X_21_
4. Participation in tourism for personal purposes (65+)	X_14_		
5. Participation in training sessions	X_15_		

The variables X_1_, X_2_, X_3_, X_5_, X_6_, X_9_, X_11_, X_13_, X_14_, X_15_, X_16_, X_17_ and X_21_ are stimulants (defining the benefit criterion for the seniors’ life situation), while the rest are considered costs. All data were sourced from Eurostat for the years 2015, 2019 and 2022.

### 3.3 Research procedure

The presented analysis was conducted in two stages. In the first stage, the specified domains, and the variables representing them, were used to construct a Synthetic Measure of Seniors’ Quality of Life (SMSQoL) in the countries of the European Union in each of the years studied: 2015, 2019 and 2022. The selection of research periods was driven by the intention to encompass both the pre-and post-COVID-19 pandemic phases, with the assumption that the interval between the chosen years would enable the observation of changes. Rankings were developed for each year using the synthetic indicator values calculated for each country. Shifts in ranking positions illustrate changes in the quality of life of seniors in each country across the analysed years. These observed changes formed the basis for testing the first research hypothesis and served as a reference point for verifying the second hypothesis.

The second stage of the analysis focused on the role of individual domains and the changes in their importance from year to year. This approach was aimed at verifying the third research hypothesis.

The Hellwig method was used to realise the specified stages of the analysis. The choice of a benchmark linear ordering method was not random. We wanted to establish a benchmark to enable the situation of each European country in each domain to be illustrated. By referring to the benchmark, it was possible to show the level of implementation of each domain in each country, in the years under study. In this way, an objective view of the living situation of senior citizens in the European Union was obtained. On the basis of the calculated values of the synthetic measures, the EU countries were classified into four groups. The shifts in the groups, in individual years, were an indicator of the course and direction of change.

### 3.4 Hellwig’s method

Thanks to linear ordering methods, it is possible to rank multidimensional objects described by characteristics selected due to the adopted research objective. The level of complexity of the phenomenon is illustrated by a measure which is a function aggregating partial information from the selected research area (79). Arranging objects in terms of the value of measures allows us to obtain a clear picture of the state of the phenomenon under study for the cases considered (e.g., EU countries). A Synthetic Measure of Seniors’ Quality of Life was constructed using linear ordering with the development pattern method (80). This method requires appropriate data preparation. In the first stage, the variables (from x_1_ to x_21_ for each EU country) are normalised so that comparability is ensured. The method chosen uses standardisation according to Equation 1:


(1) $$z_{ij}=\frac{\left(x_{ij}-{\bar{x}}_{j}\right)}{S_{j}}$$

where: *z_ij_* – standardised value of the *j*-th variable in the *i*-th object,

*x_ij_* – observed value of the *j*-th variable in the *i*-th object,

${\bar{x}}_{j}$ – mean of the *j*-th variable,

*S_j_* – standard deviation of the *j*-th variable.

As a result of variable rescaling according to the standardisation formula (Equation 1), we obtain the average value of each variable equal to zero, with the standard deviation equal to 1. In the next step of the Hellwig method, an ideal object (pattern) must be established, the values of which are selected according to the following criteria according to Equation 2:


(2) $$z_{0j}=\{\begin{array}{c} \max_{i}\left\{z_{ij}\right\}\;\mathrm{when the variable is}\;a\;\mathrm{stimulant}\\ \min_{i}\left\{z_{ij}\right\}\;\mathrm{when the variable is}\;a\;\mathrm{destimulant}\; \end{array}$$

For each object (in our case: a country), the distance from the designated reference object (pattern) is calculated. The Euclidean distance is used in this method:


(3) $$d_{i0}=\sqrt{\overset{m}{\underset{j=1}{\sum }}{\left(z_{ij}-z_{0j}\right)}^{2}},i=1,2,\ldots ,n$$

The value of the distance of each object from the pattern (Equation 3) allows us to rank these objects in order from the best (the closest to the pattern) to the worst (the furthest from the pattern) or vice versa. In order to normalise the values of the $d_{i0}$ distances obtained, as well as to obtain a measure whose rising values would indicate the development of the studied phenomenon, a synthetic measure is constructed:


(4) $$s_{i}=1-\frac{d_{i0}}{d_{0}},i=1,2,\ldots ,n$$

where: $d_{0}={\bar{d}}_{0}+2S_{d_{0}}$ ,


$${\bar{d}}_{0}=\frac{1}{n}\overset{n}{\underset{i=1}{\sum }}d_{i0}\text{,}$$

$$S_{d_{0}}=\sqrt{\frac{1}{n}\overset{n}{\underset{i=1}{\sum }}{\left(d_{i0}-{\bar{d}}_{0}\right)}^{2}}\text{.}$$

The obtained value of $s_{i}$ is the SMSQoL for each of the EU countries. Thanks to the applied formula, the measure received refers to the maximum possible distance, which is $d_{0}\text{,}$ between the ideal object and the non-ideal object. In Hellwig’s method, the measure of $s_{i}$ usually takes values in the range < 0;1>. The higher the value of the measure, the better place of a given country in the ranking (in our case, the lower the SMSQoL). When a large number of objects is considered or when one of the variables more significantly differs from the ideal object, the measure may have negative values. The ordering synthetic measure stems from the relation between the distance of a given object to an ideal object and the interval of variability of all the distances between objects and the ideal.

The value obtained using Equation 4 allows us to compare objects (in our case, EU countries). The conducted study will analyse the results of synthetic measures determined for the selected years: 2015, 2019 and 2022. However, due to the different patterns (reference levels) resulting from the actual values of the variables, the values of these measures can only be compared between objects in a specific year and not between years for a specific object.

It is possible to classify the considered objects due to differences in the values of measures. Based on the following formulas (Equation 5):


$$s_{i}\geq \bar{s}+S_{s_{i}}\text{,}$$

(5) $$\bar{s}+S_{s_{i}}>s_{i}\geq \bar{s}\text{,}$$

$$\bar{s}>s_{i}\geq \bar{s}-S_{s_{i}}$$

$$s_{i}<\bar{s}-S_{s_{i}}\text{,}$$

where: $\bar{s}=\frac{1}{n}\overset{n}{\underset{i=1}{\sum }}s_{i}\text{,}$


$$S_{s_{i}}=\sqrt{\frac{1}{n}{\left(s_{i}-\bar{s}\right)}^{2}}\;\text{.}$$

we obtain four classes of objects that are as little varied as possible within one class and as diverse as possible between classes. In the first class, there are objects with the best (highest) results of $s_{i}$ (in our case, countries with the highest level of the quality of seniors’ life). In the fourth class, characterised by the worst results, we can observe the worst level of seniors’ quality of life (81). This procedure was conducted for each year under study, and the classification results were utilised to examine changes in the composition of individual groups. Grouping is performed separately for each year, based on the SMSQoL values for each EU country in the respective year.

## 4 Results

### 4.1 SMSQoL analysis including all domains

**Figure 2:**
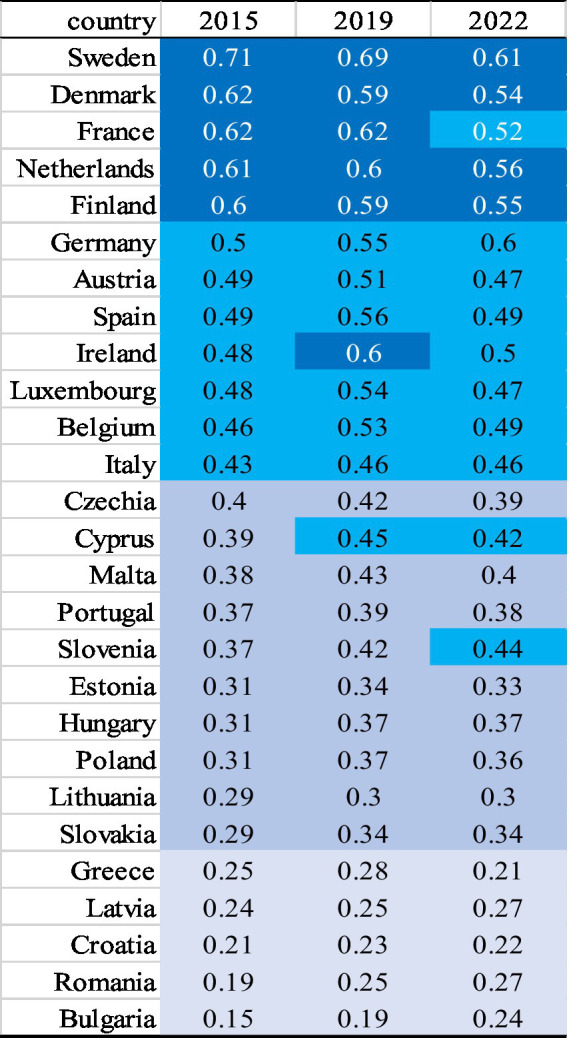
European countries ranking based on SMSQoL in each year 2015, 2019 and 2022. Source: own elaboration.

The synthetic index of the quality of life for seniors (SMSQoL) reveals inequalities among older residents of the European Union. These disparities are particularly evident through the ranking of European countries based on SMSQoL values for the years 2015, 2019, and 2022. The countries with the highest quality of life scores achieved the greatest values on our proposed measure during the analyzed years. At the same time, countries were ranked on the basis of the synthetic measures obtained in each year. The ordering and classification of countries into four groups is shown by means of a heat map (Figure 2). The darkest colour indicates the best group of countries, while the deteriorating situation is presented by the decreasing intensity of the colour. Countries are ordered according to decreasing SMSQoL values in 2015.

#### 4.1.1 Countries where the living situation of seniors is the best (Group 1—dark blue)

The living situation index for senior citizens changed over the years under consideration. The changes were generally to the detriment of the index, as its value steadily declined. In all years, Sweden achieved the best result. However, it is noticeable that the values achieved by the synthetic indicator varied in the years under study. It should be borne in mind that the values of the indicator allow for a comparison of the analysed countries in terms of quality of life in the selected year, but do not provide the possibility of comparing the country’s development in subsequent years. The situation in Denmark, the Netherlands, Finland, and France was similar. Also noteworthy is the change in the composition of this group of countries in the years under review. After being in the second group in 2015, Ireland moved to the first group in 2019, while the situation worsened in 2022, and the country was again classified in the second group. The situation was quite different in Germany, where the living situation index for seniors improved steadily. After being in the second group in 2015 and 2019, Germany climbed to the second place in the first group in 2022. Unfavourable developments can be observed in the case of France. The country was in the first group for the first two years studied, only to be classified first in the second group in 2022.

#### 4.1.2 Countries where the living situation of seniors can be described as good (above the average) (Group 2—light blue)

In the group of countries with a situation described as good, there were changes in all the years under review. This group, on each occasion, included Spain, Austria, Belgium, Luxembourg, and Italy. Ireland was added in 2015 and 2022. Germany, on the other hand, was in Group 2 in 2015 and 2019, only to climb to a higher group in 2022. France joined the group in 2022, after a decrease in the indicator compared to previous years. Being in Group 3 in 2015, Cyprus improved its position by ending up in Group 2. In the last years under review, Slovenia, classified in Group 3 in previous years, joined Group 2. The situation of the other countries in this group was stable. According to the classification adopted, the countries in this group provide good conditions for senior citizens. However, the values of the indicators, at around 50 per cent relative to the benchmark, indicate how many elements can still be improved in order for senior citizens to have a better life.

#### 4.1.3 Countries where the living situation of seniors can be considered as average (Group 3—dark grey)

The size of this group decreased in each of the years under study, while the core of the group, which consisted of the Czech Republic, Malta, Portugal, Hungary, Poland, Slovakia, Estonia and Lithuania, remained constant. Although Group 3 is the penultimate group of this classification, the values of the indicators observed in the countries belonging to this group are not satisfactory. Only 30–40% reflect the benchmark situation, which makes one reflect on the difficulties faced by senior citizens living in these countries.

#### 4.1.4 Countries where the living situation of seniors is the worst (Group 4—light grey)

The fourth group includes seniors from Greece, Romania, Croatia, Latvia and Bulgaria, where the lowest quality of life was observed. The worst situation was observed in 2015 in Bulgaria, where the Living Situation Index for seniors was 0.15, the lowest position in that year as well as in 2019, while in 2022 the worst situation was observed in Greece. The synthetic measure values for the countries in this group are very low and clearly indicate the difficult situation of seniors living in those countries. The obtained values are also the basis for the conclusion that the European Union’s efforts in the area of convergence must continue, as significant differences are still observed in relation to the quality of life of this social group.

Analysing the values of the SMSQoL indicators, it can be seen that the gap between countries is narrowing from one year to the next. However, changes in ranking positions are observed in each of the groups of countries studied. The smallest variation between countries observed in 2022 may be due to the restrictions and regulations introduced due to the COVID-19 pandemic, which were standardised across the EU.

The analysis of the synthetic measure of the quality of life (SMSQoL) revealed that several countries experienced notable improvements in the life situation of seniors over the studied years (Hypothesis 1). Sweden and Luxembourg consistently maintained high values across multiple domains, indicating stable and favourable conditions for their senior populations. Countries such as Ireland and Italy showed significant progress, particularly in the health and financial domains, reflecting effective policy interventions and support systems. However, the COVID-19 pandemic caused setbacks, particularly in social relations, which affected the overall positive trend observed in previous years. Despite these challenges, the general trend suggests that many countries succeeded in improving the life quality of their seniors before the pandemic.

The study highlighted significant disparities in the quality of life among seniors across different EU countries (Hypothesis 2). Group 1 countries, such as Sweden and Luxembourg, consistently fared better, which was reflected in higher SMSQoL values. In contrast, Group 4 countries, including Latvia and Romania, struggled with lower values and greater challenges. The persistent differences in health, finances, and social engagement across groups indicate that the diversity in the life situation of seniors has not diminished over time. These findings underscore the importance of targeted policies to address the unique needs and challenges faced by seniors in different countries, aiming to reduce these disparities.

The COVID-19 pandemic had a pronounced impact on the life situation of seniors, particularly in the social relations domain (Hypothesis 3). Many countries experienced declines in social activity indicators due to social distancing measures and lockdowns, which limited seniors’ opportunities for engagement and interaction. Financial stability and health outcomes also varied, with some countries managing the pandemic’s effects better than others. The analysis showed that countries with robust healthcare and social support systems, such as Germany and Sweden, were more resilient in cushioning the pandemic’s impact on seniors. Nevertheless, the overall trend indicates a deterioration in specific domains, particularly social relations, during the pandemic period.

### 4.2 Analysis of the quality of life of seniors in relation to domains

There is notable disparity in the values of the synthetic measure obtained in the analysed years. However, it should be remembered that the indicator values allow for comparison of the studied countries in terms of SMSQoL in a selected year, but do not enable comparison of the country’s development in subsequent years.

The analysis highlights the varying performance of different groups across multiple domains over the years. Countries in Group 1 generally showed the most consistent improvements, with Sweden often leading in health, environment, and social relations. Group 2 exhibited moderate improvements with notable progress in countries such as Italy and France. Group 3 and 4 had more variability and faced significant challenges, but countries such as Poland, Estonia and Hungary showed encouraging trends. Overall, the improvements reflect targeted efforts and policy interventions in various domains to enhance the quality of life for seniors in these countries.

In order to examine the level of development of each particular domain in each EU country in the analysed years, synthetic measures were calculated separately for each domain. The results are presented in the form of four separate graphs. The life situation of seniors was discussed for each group from the perspective of individual domains. In each figure, domains are assigned one specific colour, e.g., health – orange.

**Figure 3:**
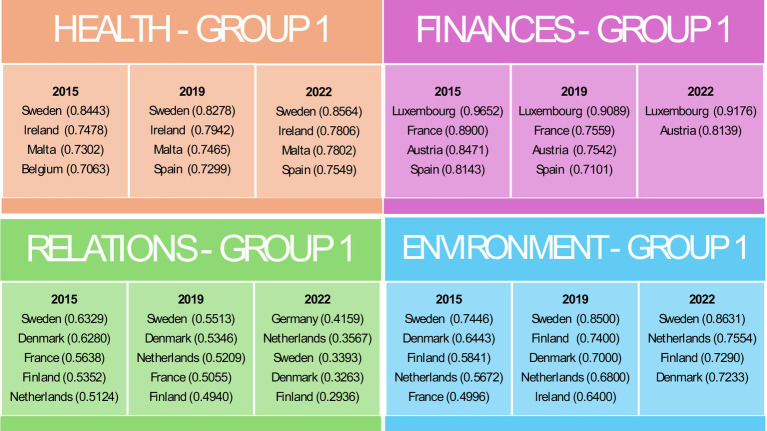
Countries belonging to Group 1 in individual domains in the years 2015, 2019 and 2022. Source: own elaboration based on Eurostat data, calculations performed in MS Excel and Statistica.

In the Health domain, Group 1 consistently demonstrated high values, particularly in countries such as Sweden and Ireland (Figure 3).

**Figure 4:**
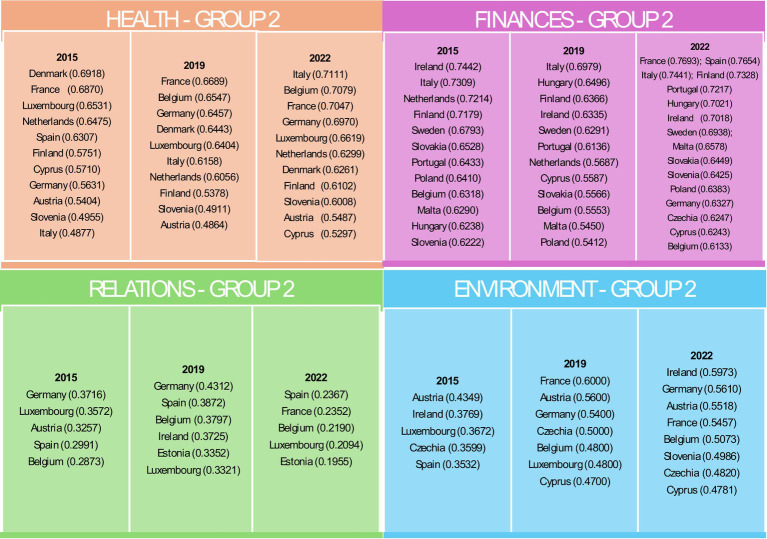
Countries belonging to Group 2 in individual domains in the years 2015, 2019 and 2022. Source: own elaboration based on Eurostat data, calculations performed in MS Excel and Statistica.

Group 2 showed moderate values with some fluctuations, especially in Denmark and France (Figure 4).

**Figure 5:**
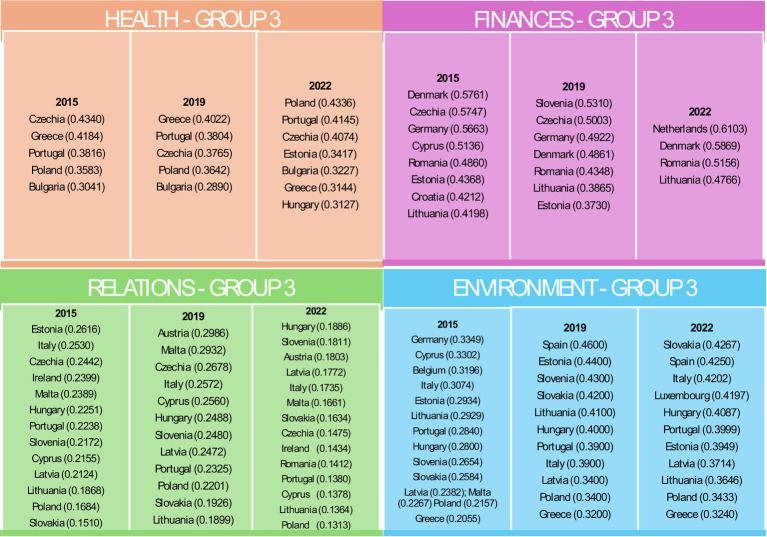
Countries belonging to Group 3 in individual domains in the years 2015, 2019 and 2022. Source: own elaboration based on Eurostat data, calculations performed in MS Excel and Statistica.

Group 3 had lower overall values but noted improvements in countries such as Poland and Estonia (Figure 5).

**Figure 6:**
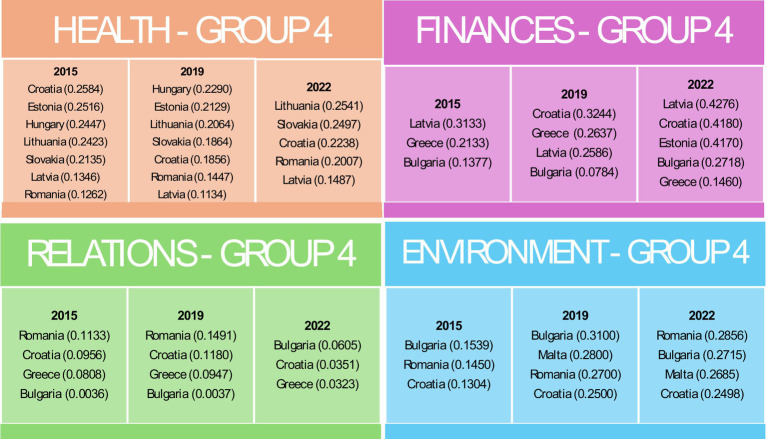
Countries belonging to Group 4 in individual domains in the years 2015, 2019 and 2022. Source: own elaboration based on Eurostat data, calculations performed in MS Excel and Statistica.

Group 4 consistently had the lowest health indicators, with significant challenges observed in Latvia and Romania (Figure 6).

In all groups, a decline in the values of indicators within the Health domain was observed in 2019, which may indicate the impact of the COVID-19 pandemic.

For the Finances domain, Luxembourg in Group 1 consistently achieved the highest financial indicators, reflecting robust economic conditions (Figure 3). Group 2 exhibited mixed performance with improvements between 2015 and 2022 in countries such as Finland, Portugal and Italy (Figure 4). Group 3 included countries such as Denmark, which faced difficulties due to high housing costs (Figure 5). Group 4, which included Greece and Bulgaria, consistently showed low financial values, indicating ongoing economic challenges (Figure 6).

The effects of restrictions associated with the COVID-19 pandemic are particularly evident in the Relationships domain. In the first group, the value of the indicator declined in both 2019 and 2022. In groups 2, 3, and 4, an initial increase in 2019 was followed by a decline, resulting in values falling below those recorded in 2015. In the Relations domain, Group 1 maintained high values and stable social activity indicators, though some decline was noted over time (Figure 3). Group 2 had moderate values but showed notable declines in 2022, likely due to the COVID-19 pandemic (Figure 4). Group 3, the most populous group, showed significant variability (Figure 5). Group 4 had the lowest values, indicating substantial challenges in social engagement and activity (Figure 6).

Regarding the Environment domain, Group 1 had high values, indicating favourable external conditions, with Sweden consistently leading (Figure 3). Group 2 was characterised by moderate values, with some countries such as Germany showing improvement over time (Figure 4). Group 3 exhibited mixed performance, with countries such as Spain showing a decline (Figures 4, 5). Group 4 had the lowest values, facing significant challenges in external conditions (Figure 6).

The analysis revealed several notable trends that significantly impact the assessment of the quality of life for seniors in European Union countries. First and foremost, it is important to highlight a group of countries that consistently achieved high scores across all analysed domains. Examples of such nations include Sweden and Luxembourg, which not only maintained high values but also showed gradual improvements. On the other hand, significant progress was observed in countries belonging to lower groups, such as Poland, Estonia, and Hungary. Despite initially being part of groups with lower indicators, these countries recorded substantial improvements, suggesting positive developments in healthcare and social policies.

The COVID-19 pandemic had a noticeable impact on social activity indicators, leading to declines in many countries due to imposed social restrictions. This phenomenon underscores the role of economic stability as a key factor influencing senior well-being, particularly evident in Luxembourg and Sweden. These countries, with their strong economic foundations, consistently performed well, highlighting the importance of economic stability in the context of senior quality of life. The improvements seen in countries such as Ireland, Germany and France also point to the effectiveness of targeted policies and interventions. Finally, the analysis revealed significant regional disparities, with Northern and Western European countries generally performing better compared to those in Eastern and Southern Europe, reflecting broader socio-economic differences within the European Union.

The recommendations based on observed trends emphasise the need for a multifaceted approach to improving the quality of life for seniors across various domains. In the Health Domain, it is crucial to strengthen healthcare systems, particularly in Group 3 and 4 countries such as Latvia, Romania, and Bulgaria, where targeted interventions and investments in healthcare infrastructure, access to medical services, and preventive measures are essential. Additionally, promoting best practices from high-performing countries such as Sweden and Ireland can drive improvements in these regions. Increasing health education and preventive care through public health campaigns focused on healthy lifestyles and senior-specific programmes, including regular health check-ups and mental health support, are also recommended to enhance overall health outcomes. In the Finances Domain, economic support for seniors should be prioritised, particularly in countries such as Greece and Bulgaria, where enhancing pension systems and implementing financial aid and subsidies for low-income seniors are necessary to improve financial security. Furthermore, launching financial literacy programmes and improving access to financial services tailored to seniors can empower them to make informed decisions and manage their finances effectively. For the Relations Domain, promoting social engagement through the establishment of community centres, regular social activities, and volunteer programmes is essential, especially in countries such as Bulgaria and Greece, where isolation among seniors is prevalent. Leveraging technology by providing digital inclusion training and organising virtual events can also help maintain social connections, particularly during times of social restrictions like those experienced during the COVID-19 pandemic. In the Environment Domain, improving living conditions through senior-friendly housing initiatives and urban planning that prioritises safe, clean, and accessible public spaces is critical for enhancing seniors’ quality of life. Additionally, enhancing environmental quality by developing green spaces and implementing stricter pollution control measures is necessary to ensure a healthy living environment for seniors, particularly in urban areas.

Cross-domain recommendations include adopting a holistic policy approach by developing integrated strategies that address multiple domains simultaneously, recognising the interconnectedness of health, finances, social engagement, and environment. Collaboration among government agencies, non-profits and the private sector is vital to create comprehensive support systems for seniors. Furthermore, data-driven decision-making should be emphasised, with systems for regular monitoring and evaluation of senior well-being across all domains to inform policy decisions and identify areas for improvement. Finally, a focus on vulnerable groups is essential, with targeted interventions for those with low incomes, chronic health conditions, and limited social support, as well as the development of culturally sensitive programmes tailored to the specific needs of different senior communities.

## 5 Discussion

The values of our proposed Senior Quality of Life indicator (SMSQoL) reveal substantial inequalities in the standard of living among older people across the European Union. These inequalities are evident across all domains – health, finances, social relations, and environment. However, financial disparities frequently underpin these broader inequalities, influencing access to healthcare, the ability to maintain social connections, and the quality of one’s living environment. Even in countries classified within the best-performing group, there remains significant room for improvement in seniors’ quality of life, particularly when considering the financial foundations that often drive disparities in other domains. Seniors represent a growing social group that increasingly influences multiple areas of the economy (2, 3, 82). The overall quality of life and well-being of European societies will be heavily shaped by how effectively these financial inequalities are addressed. The period of the COVID-19 pandemic, which is the focus of the latter years of this study, notably worsened the quality of life for seniors, with those in the best-performing countries experiencing significant declines. This trend aligns with findings from other studies (83–85). Furthermore, the findings suggest a potential convergence in the living conditions of older adults across the EU, as evidenced by the narrowing range of SMSQoL values over the analysed years. This trend may reflect the increasing effectiveness of EU policies aimed at reducing disparities, particularly for groups vulnerable to exclusion, including seniors. From the perspective of the conducted analyses, the convergence in seniors’ living conditions may indicate progress, although financial disparities continue to play a significant role in shaping these outcomes.

Authors addressing the issue of the quality of life of seniors, in various aspects, agree that the most important area of life is health, particularly considering its links with socio-economic inequalities (17, 86, 87, 101). By being physically and mentally well, seniors can maintain their independence and lead fulfilling lives (27, 52, 88, 89). In our study, in the domain of Health, the values of the synthetic measures in consecutive years are very high in the countries in the first, best group, while extremely low in the last group. The gap between the mean values of the synthetic measure is the highest for this domain each time, confirming the need for increased action in countries in Groups 3 and 4 and the implementation of measures to level the playing field within EU countries.

Next, to health, finance is one of the most important domains in the functioning of older people (7, 90). In the Finances domain, the values of the synthetic measures for the countries in the first group were the highest of all domains. For Luxembourg, the indicator had a value close to the benchmark in 2015. The variation was highest in 2019, and a reduction in the variation between countries in 2022 could indicate an improvement in the financial cohesion of seniors’ households in the EU.

The importance of social contacts and activities in healthy and happy ageing is emphasised by many authors (17, 91–93). It is also impossible to argue with the approach to social activation embodied in the idea of active ageing (51, 94, 95). The study indicates the need for intensive action in the area of social inclusion of seniors. Synthetic indicators in the area of Relationships in many countries in the years analysed show significant differences from the chosen pattern. Based on the Figures 4, 5, it is observed that as many as 17 countries belong to the two lowest classes in each year of analysis. The results of our study can be considered to be in line with the postulation of (96), who emphasise the role of maintaining social contacts in order to mitigate the effect of loneliness, and consequently improve overall well-being.

The fact that the economic situation of a country influences the living standards of its inhabitants has already been described many times (97, 98, 101). By including the Environment domain in our analysis, it was possible to consider more comprehensively the role of the state in the living comfort of seniors. The situation in this domain can be considered stable because the spreads of the synthetic measures are similar in successive years. However, this cannot be regarded as a positive phenomenon because, as in the Relationship domain, also in the Environment domain the largest number of countries in each year of the analysis can be found in the worst groups.

## 6 Conclusions

The analysis concludes that disparities exist in the quality of life among seniors across the European Union. This observed inequality necessitates the development of recommendations aimed at improving and harmonising the quality of life for seniors throughout the EU. These recommendations focus on addressing the identified disparities through targeted interventions, enhancing social engagement, improving financial security, upgrading living conditions, and adopting a comprehensive policy approach. By implementing these measures, countries can significantly advance the well-being of their ageing populations. Effective implementation will require coordinated efforts and sustained commitment from all relevant stakeholders. These recommendations are aimed at key stakeholder groups, including policy makers, non-profit organisations, healthcare providers, financial institutions and local governments. Policymakers should focus on strengthening healthcare systems in countries such as Latvia, Romania and Bulgaria by investing in medical infrastructure and prevention programmes. Not-for-profit organisations can play a key role in promoting best practices in health and social care, as well as implementing educational programmes to raise financial awareness among seniors. Healthcare providers should introduce health programmes tailored to the specific needs of seniors, providing regular check-ups and support for chronic disease management. Financial institutions should develop financial services aimed at seniors, including affordable banking and investment options, and support educational programmes on financial management. Local governments are responsible for improving the living conditions of seniors through the development of welcoming housing and safe and accessible public spaces. Collaboration between these interest groups is key to creating integrated strategies that effectively address the needs of the ageing population in different areas of life.

From an economic perspective, ageing populations should not only be viewed as a burden but also as an opportunity, particularly for reducing inequalities. In-depth analyses of seniors, their needs, attitudes or expectations can serve this purpose. While from a medical and geriatric point of view the topic seems to be well described and continuously explored, the socio-economic approach still needs to be analysed. The main challenge is the heterogeneity of the senior group, which, in our opinion, will increase in the years to come. The reason for this lies in the large differences in education, financial resources, access to new technologies or social attitudes between those currently classified as seniors and those who will soon reach this age. It should also be mentioned that our proposed SMSQoL indicator is based on domains that seniors themselves have indicated as important in their lives. The changing profile of seniors may change the domains or their importance, which will be reflected in the analysis results. This is also a very important argument, postulated by us on several occasions (source supply), in favour of the need for repeated surveys. The indicators used to describe the domains, despite their arbitrary selection dictated by the availability of data, allowed us to obtain an objective picture of the living situation of European seniors in particular years.

The continuation of the research can be carried out on several levels. The first relates to the possibility of applying the research path we presented to other indicators selected to describe the domains. Another relates to the determination of the reference level of the variables in the individual domains, in such a way that the pattern, or object of reference, is the same in each of the years studied. Research on the quality of life of seniors, conducted in separate age groups, could be of great value to the results presented in this article. Such an approach may contribute to a better understanding of the social group of seniors, despite its considerable diversity. Aligned with the theory of intersectionality proposed by Crenshaw (99) in the context of women of colour, ageing experiences are neither uniform nor confined to quality of life (QoL) issues. Instead, they are influenced by the intersections of race, gender, class, and other identity dimensions. We underscore the importance of research that examines diverse and intersecting social contexts to deepen our understanding of inequalities associated with aging.

## Data Availability

The raw data supporting the conclusions of this article will be made available by the authors, without undue reservation.
